# Eosinophilic bioactivities in severe asthma

**DOI:** 10.1186/s40413-016-0112-5

**Published:** 2016-06-27

**Authors:** Tara F. Carr, Sergejs Berdnikovs, Hans-Uwe Simon, Bruce S. Bochner, Lanny J. Rosenwasser

**Affiliations:** Children’s Mercy Hospital, Kansas City, Missouri USA; University of Arizona, Tucson, Arizona USA; Northwestern University Feinberg School of Medicine, Chicago, Illinois USA; Institute of Pharmacology, University of Bern, Bern, Switzerland

**Keywords:** Severe asthma, Eosinophils, Eosinophilia, Bioactivities, Biotherapeutics

## Abstract

Asthma is clearly related to airway or blood eosinophilia, and asthmatics with significant eosinophilia are at higher risk for more severe disease. Eosinophils actively contribute to innate and adaptive immune responses and inflammatory cascades through the production and release of diverse chemokines, cytokines, lipid mediators and other growth factors. Eosinophils may persist in the blood and airways despite guidelines-based treatment. This review details eosinophil effector mechanisms, surface markers, and clinical outcomes associated with eosinophilia and asthma severity. There is interest in the potential of eosinophils or their products to predict treatment response with biotherapeutics and their usefulness as biomarkers. This is important as monoclonal antibodies are targeting cytokines and eosinophils in different lung environments for treating severe asthma. Identifying disease state-specific eosinophil biomarkers would help to refine these strategies and choose likely responders to biotherapeutics.

## Background

Eosinophils are terminally differentiated granulocytes that play a role in innate host defense against pathogens, particularly parasites and viruses. Eosinophils damage both pathogenic and host cells through the release of toxic granule proteins and reactive oxygen species. Far from maintaining a bystander role, however, eosinophils actively contribute to innate and adaptive inflammatory cascades through the production and release of diverse chemokines, cytokines, lipid mediators and other growth factors. Through these effector mechanisms, eosinophils can influence tissue specific function. Asthma, a chronic airways disease, is often related to airway or blood eosinophilia. Importantly, asthmatics with significant eosinophilia are at higher risk for more severe disease. Strategies for identifying and treating these individuals will provide much needed progress toward personalized medical care. This review details eosinophil effector mechanisms, surface markers, and clinical outcomes associated with eosinophilia and asthma severity.

### Key points

- Eosinophils contain highly charged proteins in their granules, mediating toxicity toward pathogens and tissues, and produce a variety of inflammatory proteins which further contribute to tissue pathology
- Eosinophil accumulation in the airways in severe asthma correlate with markers of local tissue and extracellular matrix (ECM) remodeling
- Eosinophilia is a marker of severe asthma and those at risk for more frequent exacerbations

#### Unmet needs

- Understanding phenotypic and functional plasticity of eosinophils in the context of the changing or altered lung microenvironment occurring within different asthma endotypes and severity
- Discovery of how the phenotype and function of eosinophils contribute to asthma exacerbations
- Identification of novel eosinophil-restricted biomarkers, both internal and external, with potential to be targeted for therapeutic intervention with specific biotherapeutics and monoclonal antibodies.

## Effector and pro-inflammatory mechanisms of eosinophils

**Fig. 1:**
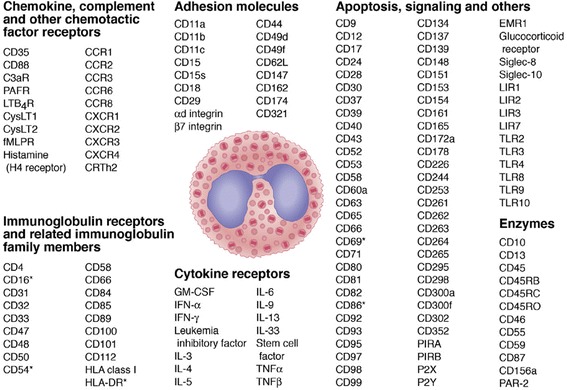
Surface molecules expressed by human eosinophils. There is some overlap among categories for some of these proteins. Common names for chemokine (CC and CXC) receptors, toll-like receptors (TLRs), and others were sometimes used instead of the CD names because of the greater use and familiarity among most readers of the former. The asterisk indicates activated eosinophils. C3aR, C3a receptor; CysLT, cysteinyl leukotriene receptor type; EMR1, epidermal growth factor–like module containing mucin-like hormone receptor-like 1; fMLPR, formyl-methionyl-leucyl-phenylalanine receptor; GM-CSF, granulocyte-macrophage colonystimulating factor; IFN, interferon; IL, interleukin; LIR, leukocyte immunoglobulin-like receptor; LTB4R, leukotriene B4 receptor; P2X and P2Y, two types of purinergic receptors; PAFR, platelet activating factor receptor; PIR, paired immunoglobulin-like receptor; TNF, tumor necrosis factor. (Courtesy of Jacqueline Schaffer, MAMS, Chicago, IL.). Reproduced with permission [27]

**Fig. 2:**
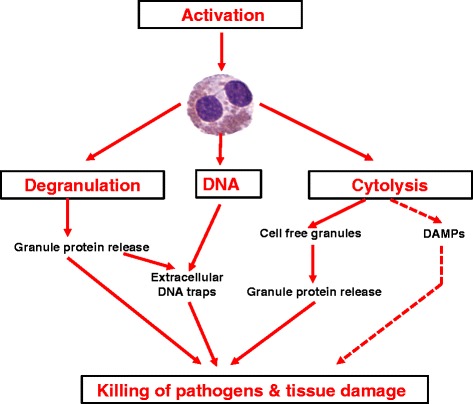
Eosinophil effector mechanisms following eosinophil activation. Among other pro-inflammatory mediators, eosinophils release granular proteins and mitochondrial DNA. As a consequence, eosinophil granule proteins mediate toxicity toward pathogens or host cells, either alone and with extracellular DNA traps. Cytolysis is associated with massive granule protein secretion. Moreover, cytolysis most likely results in the release of damage-associated molecular pattern molecules (DAMPs), which are known to trigger inflammatory responses. The release of DAMPs from cytolytic eosinophils, however, remains to be demonstrated; hence, this pathway is indicated with dashed arrows

Eosinophils are terminally differentiated granulocytic effector cells that produce and store biologically active molecules, including the cytotoxic proteins major basic protein (MBP), eosinophil peroxidase (EPX), eosinophil cationic protein (ECP), and eosinophil derived neurotoxin (EDN), lipid mediators, chemotactic peptides, as well as cytokines (Fig. 1) [1, 2]. They are believed to play an important role in the innate immune system by contributing to host resistance to parasites, particularly helminths, but also antimicrobial activities toward bacterial, viral and fungal pathogens [3]. These pathogens can be killed by granule proteins released from activated eosinophils [4]. However, the eosinophil-derived granule proteins are not only toxic to pathogens but also to other cells within immune responses, causing tissue damage and consequently organ dysfunction (Fig. 2). MBP is expressed as two homologs, MBP-1 and MBP-2; MBP-1 has a markedly basic pH and is directly toxic to host and parasite cells [5]. EDN exerts antiviral effects through RNase activity [6]. Besides granule proteins, eosinophil reactive oxygen species have also been shown to damage cells and tissues. EPX catalyzes the development of reactive oxygen species in the presence of hydrogen peroxide. Not only the cytotoxic activity itself, but also the interactions with other immune cells, enhanced the notion of a pro-inflammatory role for the eosinophil [7]. It could be demonstrated that MBP induces histamine release by both mast cells and basophils [8]. Furthermore, EPX in combination with H_2_O_2_ and halides was shown to induce mast cell secretion [9].

To release granule proteins and to produce toxic oxygen radicals, eosinophils need to be activated (Fig. 2). Following priming with eosinophil hematopoietins, such as IL-5 and GM-CSF, eosinophils exhibit an increased capacity to respond to other inflammatory mediators [10]. Adhesion molecule engagement can also stimulate eosinophil activation [11]. Activation of the mitogen-activated protein kinase (MAPK) pathway is central for degranulation [12]. Granule proteins can be detected in tissues following eosinophil degranulation, sometimes even in the absence of sustained eosinophil infiltration [13], suggesting not only little degradation but also limited toxicity under such conditions. Extracellular granule proteins can be detected in association with collagen fibers, within DNA traps and extracellular free granules (Fig. 2) [14]. MBP has been shown to occur as an amyloid structure within flame figures of patients with eosinophilic dermatitis [15] and eosinophil DNA traps have been demonstrated in multiple eosinophilic diseases, including eosinophilic asthma [16]. Although the DNA scaffold clearly increases the toxicity of granule proteins against bacteria, it remains unclear whether eosinophil DNA traps mediate or limit immunopathology [17]. Extracellular free granules are believed to be the result of eosinophil cytolysis, a non-apoptotic cell death occurring as a consequence of utmost eosinophil activation [18] (Fig. 2). The molecular mechanism leading to eosinophil cytolysis, however, is not known.

The eosinophil’s granule proteins exhibit different toxicity when tested in vitro. While EDN is only marginally toxic, MBP, EPO and ECP are toxic to a variety of tissues, including heart, brain, skin, and bronchial epithelium [4]. ECP has been shown to be 8–10 times more potent than MBP [19]. Recently, however, it has been demonstrated that the toxicity of MBP is regulated by aggregation [15]. Following degranulation, MBP forms functional amyloid structures exhibiting high toxicity. The continuation of the aggregation process, however, is associated with a loss of toxicity explaining the little damage seen in tissues containing large MBP deposits. MBP can stimulate histamine and leukotriene C4 release from human basophils and can activate mast cells. Interestingly, heparin, a product of mast cells, can rapidly inhibit MBP toxicity [15]. Therefore, MBP toxicity is not stable and depends on polymorphic self-association pathways. Taken together, first insights have been obtained on how the non-selective mechanism of MBP toxicity toward host cells, such as bronchial epithelial cells in asthma, is regulated.

In addition to the direct tissue toxic effect of eosinophil activation and degranulation, eosinophils can contribute to inflammatory pathways through their capacity to synthesize and secrete a remarkable number of pro-inflammatory cytokines and chemokines [20] (Fig. 1). For example, eosinophils may produce type-2 pathway factors such as interleukin (IL)-4, IL-5, IL-13, and IL-25, and may have been shown to do so in the airways of asthmatics [21]. Chemokines such as CCL5/RANTES, CCL11/eotaxin and CCL3 are secreted by eosinophils and can recruit leukocytes to the site of eosinophilic inflammation. Alternately, following allergen challenge, airway eosinophils have been shown to express GM-CSF and CXCL8/IL-8 [22] thereby inducing neutrophil recruitment. Eosinophils may contribute to airway remodeling in severe asthma through release of transforming growth factor β-1 [23, 24]. Interferon-gamma, a type-1 cytokine secreted by eosinophils, can also potently activate eosinophils [25], and is elevated in the serum of some acute severe asthmatic patients [26], underscoring the importance of these pathways in severe asthma.

The pleiotropic effects of eosinophils therefore include host defense, host tissue damage, regulation and potentiation of inflammatory cascades, each of which may contribute to asthma severity. Therapies that target eosinophils may therefore help control diseases associated with eosinophil-mediated tissue damage and inflammation [7, 27].

## Markers on eosinophils

Like all leukocytes, the eosinophil displays a wide range of cell surface proteins, glycans, and lipids (see Fig. 1) [2, 28]. Functions associated with this phenotype include adhesion, migration, proliferation, activation and promotion of survival and death. Some cell surface receptors are G-protein coupled receptors (e.g., chemokine receptors), some are members of the immunoglobulin gene superfamily or have enzymatic activity, while others allow the eosinophil to interact with various soluble mediators released by other immune-related proteins such as cytokines and immunoglobulins. Eosinophils are also equipped with certain intracellular receptors that regulate function (e.g., some toll-like receptors and the glucocorticoid receptor). The only known surface structure that is completely unique to the eosinophil is epidermal growth factor–like module containing mucin-like hormone-like receptor 1 (EMR1) [29, 30]. Other cell surface structures are relatively specific for eosinophils, but are also expressed on mast cells and basophils, such as CCR3 (the receptor for eotaxins), CRTh2 (the receptor for prostaglandin D2), the IL-5 receptor, and Siglec-8. Glycomic analysis of materials derived from cell lysates showed sizable amounts of terminal N-acetylglucosamine containing structures in both eosinophils and basophils, while mast cells have more in the way of sialylated terminal glycans [31].

Markers of eosinophil activation may differ among the atopic diseases, both by site and severity. Identification of markers on tissue resident eosinophils has been limited by the availability of adequate tissue for this analysis, particularly in severe asthma. An extensive review on the role of allergic biomarkers of asthma and other atopic disease was published recently by the World Allergy Organization [32].

Airway eosinophilia occurs in approximately 50 % of severe asthma patients, and is especially prominent in severe asthmatics with Type 2-like late onset disease [33]. The number of eosinophils in sputum can be used as a marker of disease severity, with strong associations between airway eosinophilia and severity of disease symptoms, worsened lung function, and incidence of fatal asthma [33, 34]. Eosinophil numbers in the airways correlate with tissue biomarkers such as periostin, fractional exhaled nitric oxide expression of chemokine CCL26 (eotaxin-3), and thickening of the reticular basement membrane with deposition of extracellular matrix (ECM) proteins, specifically laminin and tenascin [35–37]. Airway eosinophils in asthma exhibit a hyperadhesive phenotype towards various ECM components, and are thought to play an active role in lung tissue remodeling processes and thickening of the reticular basement membrane through their release of TGF-β and proteases [38, 39].

Following recruitment into the airways, there are certain predictable and characteristic changes in the surface phenotype of the eosinophil reflecting their transendothelial and transepithelial migration and interactions with the tissue and ECM, such as shedding of L-selectin (CD62L), CD31, the IL-5 receptor and CD162, upregulation and/or enhanced function of CD11b, CD11c, CD11d, CD35, CD44 (the receptor for hyalouronic acid), CD66 and CD81, and the de novo appearance of HLA-DR, ICAM-1 and CD69 [40, 41]. Upregulation of an allosterically active form of integrin CD11b is particularly characteristic of the airway eosinophil phenotype in asthma, mediating enhanced adhesion and migration of eosinophils to fibrinogen and diverse ECM ligands [42]. CD11b was also recently established as a primary adhesive and pro-migratory receptor for periostin [43]. An interesting possibility has been proposed that in patients with severe eosinophilic asthma, in situ maturation of CD34^+^ and CD125^+^ (IL-5Rα) eosinophil progenitors rather than recruitment of mature cells could also contribute to persistent airway eosinophilia [44, 45], potentially governed by IL-5 produced by group 2 innate lymphoid cells [46].

By now, it is widely recognized that asthma is a highly heterogeneous disease. Several endotypes potentially exist even in forms of disease characterized by eosinophil presence in the airways, where diverse factors could dictate specific eosinophil phenotypes [47]. Our understanding of eosinophil phenotypic plasticity across different asthma endotypes is currently lacking. Identifying disease state-specific eosinophil biomarkers would not only allow for better understanding of the nature of eosinophil-tissue relationship, but also would help to refine our strategies for targeting these cells in different lung microenvironments.

## Eosinophils, severe asthma and allergic inflammation

Eosinophils are present in the blood and some tissues of healthy individuals and are increased in numbers in a variety of disease states. Systemic diseases, such as parasitic infection, drug allergy, vasculitis, or malignancy, can present with blood or organ-specific eosinophilia. Similarly, eosinophilia can be a marker of many diseases of the airway, such as chronic sinusitis, chronic obstructive pulmonary disease, hypersensitivity pneumonitis, acute and chronic eosinophilic pneumonia, and of course, asthma. Indeed, elevated eosinophil levels are seen in approximately half of individuals with asthma [48–51]. Here we discuss the relationship between atopy and eosinophilia as it may pertain to severe asthma.

Atopy is a prominent feature of some asthma phenotypes. Environmental allergens, such as *Dermatophagoides* spp., *Alternaria alternata*, pollens and pet dander have been implicated in the development, or severity, of asthma in epidemiologic studies [52, 53]. These allergens presumably exert their effects through activation of mast cells and basophils. Mast cells are bone marrow derived cells of the innate immune system which are induced by stem cell factor and IL-3, mature and reside in tissues, and can proliferate in tissues after maturation. Mast cell granules contain pre-formed mediators including histamine, tryptase, and variably other enzymes such as chymase and carboxypeptidase. Allergen-specific IgE antibodies noncovalently bind to the high affinity IgE receptor (FcεRI) on the surface of tissue resident mast cells. Mast cells can be activated by cross-linking of those FcεRI molecules upon exposure of the mast cell to the offending antigen. This event initiates signalling cascades within the mast cell involving protein tyrosine kinases. Three main pathways predominate. The first involves phosphatidylinostol bisphosphate catabolism and activation of protein kinase C, which together facilitate mast cell degranulation and release of the aforementioned preformed mediators. The mast cell activation cascade also activates phospolipase A_2_, which induces development of arachadonic acid, and the subsequent production of the lipid mediators prostaglandin D2 and the cysteinyl-leukotrienes. Finally, activation of the kinase cascades leads to nuclear translocation of transcription factors which stimulate gene expression and protein production of cytokines such as IL-4, IL-5, IL-13 and tumor necrosis factor. The IL-5 released stimulates bone marrow production and release of eosinophils, which are then recruited to tissues via ICAM-1, P-selectin and VCAM-1. Type-2 helper CD4+ T lymphocytes are recruited, and chronically contribute proinflammatory mediators which potentiate this cycle.

As discussed earlier in this chapter, eosinophils can cause direct toxic effects on host tissues and promote inflammatory cascades through release of a variety of inflammatory mediators. These effects are reflected in clinical outcomes, particularly severity of asthma and risk of exacerbation. Severe asthma is defined as asthma that requires treatment with high dose inhaled corticosteroids (ICS) plus a second controller for the previous year, and/or systemic corticosteroids for at least half of the previous year, to prevent it from becoming ‘uncontrolled’ or which remains ‘uncontrolled’ despite this therapy. Uncontrolled asthma is defined as the presence at least one of the following characteristics: persistently poor symptom control, two or more exacerbations requiring bursts of systemic corticosteroids in the preceding year, at least one serious exacerbation requiring hospitalization in the previous year, or chronic airflow limitation of FEV1 < 80 % predicted with FEV1/FVC ratio less than the lower limit of normal [54].

An analysis using the National Health and Nutrition Examination Survey, an annual cross-sectional survey of the US general population, revealed that individuals with asthma and blood eosinophil count greater than 300 cells per microliter were more likely to report asthma attacks [55]. Similarly, adults with higher blood eosinophil counts seem to have more frequent exacerbations than those with low eosinophil counts [56]. In the National Institutes of Health-sponsored Severe Asthma Research Program (SARP), which enrolled and carefully assessed large cohorts of mild, moderate, and severe asthmatic adults and children, eosinophilic and other cellular markers were assessed in relationship to disease outcomes. Those individuals with significant sputum eosinophilia, often in the presence of sputum neutrophilia, had more severe asthma. Importantly, these groups also had increased medication use, bursts of systemic corticosteroids, and hospitalizations [57, 58]. Reduction of eosinophil levels in blood and sputum is also related to fewer exacerbations and less health care utilization for asthma [59, 60]. However, in some severe asthmatics, high eosinophil levels can persist despite the use of high dose controller medications, including corticosteroids [61].

Importantly, eosinophilia is a marker of beneficial response to corticosteroid therapy [61–64]. Therefore, identification of asthmatics with significant eosinophilic inflammation is an important step towards practicing personalized, or precision, medicine. Eosinophilia can be present in the airway lumen, bronchial walls, and blood, however levels in these compartments do not always correlate. Cell counts and gene expression patterns in the sputum can accurately identify steroid-responders [62, 64]; however, induced sputum collection and measurement is time consuming, labor-intensive, and not available for routine use [65]. Blood eosinophil measurements, while simple and widely available, are a good predictor of steroid response [59, 66]. However, blood eosinophil levels can fluctuate throughout the day, with higher levels in the evening, impacting accuracy of measurements. ECP can be used as a biomarker of eosinophilic inflammation, and can be measured in both blood and airway compartments. In plasma, ECP may be a time sensitive marker of eosinophil activation [67].

Anti-IL-5 monoclonal antibodies, mepolizumab, and reslizumab are available for the treatment of severe eosinophilic asthma. Phase three studies supported its use for reduction of exacerbation frequency and steroid sparing effect [68–70]. Long term effects on airway remodeling remain to be seen.

## Conclusions

Eosinophils can affect airway biology as both a source of epithelial damage and airway remodeling in asthma. As such eosinophils contribute to the severity of asthma and may persist despite guidelines-based treatment. Eosinophils also may act as biomarkers for severity of asthma and may also identify the response to treatment and control for severe asthma. Eosinophils or their products are of great interest for potential usefulness in predicting treatment response and as biomarkers. This is especially important since new and expensive biotherapeutic treatments (monoclonal antibodies) are being directed towards eosinophils in the treatment of severe asthma.

## Abbreviations

ECM, extra cellular matrix; ECP, eosinophil cationic protein; EDN, eosinophil derived neurotoxin; EPX/EPO, eosinophil peroxidase; MAPK, mitogen activated protein kinase; MBP, major basic protein

## References

[1] Rothenberg ME, Hogan SP (2006). The eosinophil. Annu Rev Immunol.

[2] Bochner BS (2015). Novel Therapies for Eosinophilic Disorders. Immunol Allergy Clin North Am.

[3] Rosenberg HF, Dyer KD, Foster PS (2013). Eosinophils: changing perspectives in health and disease. Nat Rev Immunol.

[4] Acharya KR, Ackerman SJ (2014). Eosinophil granule proteins: Form and function. J Biol Chem.

[5] Plager DA, Loegering DA, Weiler DA (1999). A novel and highly divergent homolog of human eosinophil granule major basic protein. J Biol Chem.

[6] Domachowske JB, Dyer KD, Bonville CA, Rosenberg HF (1998). Recombinant human eosinophil-derived neurotoxin/RNase 2 functions as an effective antiviral agent against respiratory syncytial virus. J Infect Dis.

[7] Fulkerson PC, Rothenberg ME (2013). Targeting eosinophils in allergy, inflammation and beyond. Nat Rev Drug Discov.

[8] Cramer O’Donnell M, Ackerman SJ, Gleich GJ, Thomas LJ (1983). Activation of basophil and mast cell histamine release by eosinophil granule major basic protein. J Exp Med.

[9] Henderson WR, Chi EY, Klebanoff SJ (1980). Eosinophil peroxidase-induced mast cell secretion. J Exp Med.

[10] Takafuji S, Bischoff SC, DeWeck AL, Dahinden CA (1991). IL-3 and IL-5 prime normal human eosinophils to produce leukotriene C4 in response to soluble agonists. J Immunol.

[11] Yoon J, Ponikau JU, Lawrence C, Kita H (2008). Innate anti-fungal immunity of human eosinophils mediated by a β2-integrin, CD11b. J Immunol.

[12] Adachi T, Choudhury BK, Stafford S, Sur S, Alam R (2000). The differential role of extracellular signal-regulated kinases and p38 mitogen-activated protein kinase in eosinophil functions. J Immunol.

[13] Wright BL, Leiferman KM, Gleich GJ (2011). Eosinophil granule protein localization in eosinophilic endomyocardial disease. N Engl J Med.

[14] Simon D, Hoesli S, Roth N, Staedler S, Yousefi S, Simon HU (2011). Eosinophil extracellular DNA traps in skin diseases. J Allergy Clin Immunol.

[15] Soragni A, Yousefi S, Stoeckle C, Soriaga AB, Sawaya MR, Kozlowski E (2015). Toxicity of eosinophil MBP is repressed by intracellular crystallization and promoted by extracellular aggregation. Mol Cell.

[16] Dworski R, Simon HU, Hoskins A, Yousefi S (2011). Eosinophil and neutrophil extracellular DNA traps in human allergic asthmatic airways. J Allergy Clin Immunol.

[17] Yousefi S, Gold JA, Andina N, Lee JJ, Kelly AM, Kozlowski E (2008). Catapult-like release of mitochondrial DNA by eosinophils contributes to antibacterial defense. Nat Med.

[18] Erjefält JS, Andersson M, Greiff L, Korsgren M, Gizycki M, Jeffery PK (1998). Cytolysis and piecemeal degranulation as distinct modes of activation of airway mucosal eosinophils. J Allergy Clin Immunol.

[19] Ackerman SJ, Gleich GJ, Loegering DA, Richardson BA, Butterworth AE (1985). Comparative toxicity of purified human eosinophil granule cationic proteins for schistosomula of Schistosoma mansoni. Am J Trop Med Hyg.

[20] Davoine F, Lacy P (2014). Eosinophil cytokines, chemokines, and growth factors: emerging roles in immunity. Front Immunol.

[21] Ying S, Humbert M, Barkans J (1997). Expression of IL-4 and IL-5 mRNA and protein product by CD4+ and CD8+ T cells, eosinophils, and mast cells in bronchial biopsies obtained from atopic and nonatopic (intrinsic) asthmatics. J Immunol.

[22] Yousefi S, Hemmann S, Weber M (1995). IL-8 is expressed by human peripheral blood eosinophils. Evidence for increased secretion in asthma. J Immunol.

[23] Ohno I, Lea RG, Flanders KC (1992). Eosinophils in chronically inflamed human upper airway tissues express transforming growth factor beta 1 gene (TGF beta 1). J Clin Invest.

[24] Minshall EM, Leung DY, Martin RJ (1997). Eosinophil-associated TGF-beta1 mRNA expression and airways fibrosis in bronchial asthma. Am J Respir Cell Mol Biol.

[25] Valerius T, Repp R, Kalden JR, Platzer E (1990). Effects of IFN on human eosinophils in comparison with other cytokines. A novel class of eosinophil activators with delayed onset of action. J Immunol.

[26] Corrigan CJ, Kay AB (1990). CD4 T-lymphocyte activation in acute severe asthma. Relationship to disease severity and atopic status. Am Rev Respir Dis.

[27] Radonjic-Hoesli S, Valent P, Klion AD, Wechsler ME, Simon HU (2015). Novel targeted therapies for eosinophil-associated diseases and allergy. Annu Rev Pharmacol Toxicol.

[28] von Gunten S, Ghanim V, Valent P, Bochner BS, Adkinson NF, Bochner BS, Busse WW, Holgate ST, Lemanske R, O’Hehir R (2014). Appendix A. CD molecules. Middleton’s Allergy Principles and Practice.

[29] Hamann J, Koning N, Pouwels W, Ulfman LH, van Eijk M, Stacey M (2007). EMR1, the human homolog of F4/80, is an eosinophil-specific receptor. Eur J Immunol.

[30] Legrand F, Tomasevic N, Simakova O, Lee CC, Wang Z, Raffeld M (2014). The eosinophil surface receptor epidermal growth factor-like module containing mucin-like hormone receptor 1 (EMR1): a novel therapeutic target for eosinophilic disorders. J Allergy Clin Immunol.

[31] North SJ, von Gunten S, Antonopoulos A, Trollope A, MacGlashan DW, Jang-Lee J (2012). Glycomic analysis of human mast cells, eosinophils and basophils. Glycobiology.

[32] Metcalfe DD, Pawankar R, Ackerman SJ (2016). Biomarkers of the involvement of mast cells, basophils and eosinophils in asthma and allergic diseases. World Allergy Organ J.

[33] Trejo Bittar HE, Yousem SA, Wenzel SE (2015). Pathobiology of severe asthma. Annu Rev Pathol.

[34] Miranda C, Busacker A, Balzar S, Trudeau J, Wenzel SE (2004). Distinguishing severe asthma phenotypes: role of age at onset and eosinophilic inflammation. J Allergy Clin Immunol.

[35] Arron JR, Izuhara K (2015). Asthma biomarkers: what constitutes a 'gold standard'?. Thorax.

[36] Larose MC, Chakir J, Archambault AS, Joubert P, Provost V, Laviolette M (2015). Correlation between CCL26 production by human bronchial epithelial cells and airway eosinophils: Involvement in patients with severe eosinophilic asthma. J Allergy Clin Immunol.

[37] Phipps S, Flood-Page P, Menzies-Gow A, Ong YE, Kay AB (2004). Intravenous anti-IL-5 monoclonal antibody reduces eosinophils and tenascin deposition in allergen-challenged human atopic skin. J Invest Dermatol.

[38] Flood-Page P, Menzies-Gow A, Phipps S, Ying S, Wangoo A, Ludwig MS (2003). Anti-IL-5 treatment reduces deposition of ECM proteins in the bronchial subepithelial basement membrane of mild atopic asthmatics. J Clin Invest.

[39] Lee JJ, Jacobsen EA, McGarry MP, Schleimer RP, Lee NA (2010). Eosinophils in health and disease: the LIAR hypothesis. Clin Exp Allergy.

[40] Na HJ, Hamilton RG, Klion AD, Bochner BS (2012). Biomarkers of eosinophil involvement in allergic and eosinophilic diseases: review of phenotypic and serum markers including a novel assay to quantify levels of soluble Siglec-8. J Immunol Methods.

[41] Johansson MW (2014). Activation states of blood eosinophils in asthma. Clin Exp Allergy.

[42] Barthel SR, Jarjour NN, Mosher DF, Johansson MW (2006). Dissection of the hyperadhesive phenotype of airway eosinophils in asthma. Am J Respir Cell Mol Biol.

[43] Johansson MW, Annis DS, Mosher DF (2013). alpha(M)beta(2) integrin-mediated adhesion and motility of IL-5-stimulated eosinophils on periostin. Am J Respir Cell Mol Biol.

[44] Dorman SC, Efthimiadis A, Babirad I, Watson RM, Denburg JA, Hargreave FE (2004). Sputum CD34 + IL-5Ralpha + cells increase after allergen: evidence for in situ eosinophilopoiesis. Am J Respir Crit Care Med.

[45] Fanat AI, Thomson JV, Radford K, Nair P, Sehmi R (2009). Human airway smooth muscle promotes eosinophil differentiation. Clin Exp Allergy.

[46] Smith SG, Chen R, Kjarsgaard M, Huang C, Oliveria JP, O'Byrne PM (2015). Increased numbers of activated group 2 innate lymphoid cells in the airways of patients with severe asthma and persistent airway eosinophilia. J Allergy Clin Immunol.

[47] Fajt ML, Wenzel SE (2015). Asthma phenotypes and the use of biologic medications in asthma and allergic disease: the next steps toward personalized care. J Allergy Clin Immunol.

[48] Zhang JY, Wenzel SE (2007). Tissue and BAL based biomarkers in asthma. Immunol Allergy Clin North Am.

[49] Wenzel SE, Schwartz LB, Langmack EL (1999). Evidence that severe asthma can be divided pathologically into two inflammatory subtypes with distinct physiologic and clinical characteristics. Am J Respir Crit Care Med.

[50] Douwes J, Gibson P, Pekkanen J, Pearce N (2002). Non-eosinophilic asthma: importance and possible mechanisms. Thorax.

[51] Schleich FN, Manise M, Sele J, Henket M, Seidel L, Louis R (2013). Distribution of sputum cellular phenotype in a large asthma cohort: predicting factors for eosinophilic vs neutrophilic inflammation. BMC Pulm Med.

[52] Sporik R, Holgate ST, Platts-Mills TA, Cogswell JJ (1990). Exposure to house-dust mite allergen (Der p I) and the development of asthma in childhood. A prospective study. N Engl J Med.

[53] Taussig LM, Wright AL, Holberg CJ, Halonen M, Morgan WJ, Martinez FD (2003). Tucson Children's Respiratory Study: 1980 to present. J Allergy Clin Immunol.

[54] Chung KF, Wenzel SE, Brozek JL, Bush A, Castro M (2014). International ERS/ATS guidelines on definition, evaluation and treatment of severe asthma. Eur Respir J.

[55] Tran TN, Khatry DB, Ke X, Ward CK, Gossage D (2014). High blood eosinophil count is associated with more frequent asthma attacks in asthma patients. Ann Allergy Asthma Immunol.

[56] Zeiger RS, Schatz M, Li Q (2014). High blood eosinophil count is a risk factor for future asthma exacerbations in adult persistent asthma. J Allergy Clin Immunol Pract.

[57] Moore WC, Fitzpatrick AM, Li X (2013). Clinical heterogeneity in the severe asthma research program. Ann Am Thorac Soc.

[58] Moore WC, Hastie AT, Li X (2014). Sputum neutrophil counts are associated with more severe asthma phenotypes using cluster analysis. J Allergy Clin Immunol.

[59] Pavord ID, Korn S, Howarth P (2012). Mepolizumab for severe eosinophilic asthma (DREAM): a multicentre, double-blind, placebo-controlled trial. Lancet.

[60] Green RH, Brightling CE, McKenna S (2002). Asthma exacerbations and sputum eosinophil counts: a randomised controlled trial. Lancet.

[61] Arron JR, Choy DF, Scheerens H, Matthews JG (2013). Noninvasive biomarkers that predict treatment benefit from biologic therapies in asthma. Ann Am Thorac Soc.

[62] Peters MC, Mekonnen ZK, Yuan S, Bhakta NR, Woodruff PG, Fahy JV (2014). Measures of gene expression in sputum cells can identify TH2-high and TH2-low subtypes of asthma. JACI.

[63] Baines KJ, Simpson JL, Wood LG, Scott RJ, Gibson PG (2011). Transcriptional phenotypes of asthma defined by gene expression profiling of induced sputum samples. J Allergy Clin Immunol.

[64] Baines KJ, Simpson JL, Wood LG (2014). Sputum gene expression signature of 6 biomarkers discriminates asthma inflammatory phenotypes. J Allergy Clin Immunol.

[65] Peters SP (2011). Counterpoint: Is measuring sputum eosinophils useful in the management of severe asthma? No, not for the vast majority of patients. Chest.

[66] Castro M, Wenzel SE, Bleecker ER (2014). Benralizumab, an anti-interleukin 5 receptor α monoclonal antibody, versus placebo for uncontrolled eosinophilic asthma: a phase 2b randomised dose-ranging study. Lancet Respir Med.

[67] Björk A, Venge P, Peterson CG (2000). Measurements of ECP in serum and the impact of plasma coagulation. Allergy.

[68] Bel EH, Wenzel SE, Thompson PJ (2014). Oral glucocorticoid-sparing effect of mepolizumab in eosinophilic asthma. N Engl J Med.

[69] Ortega HG, Liu MC, Pavord ID (2014). Mepolizumab treatment in patients with severe eosinophilic asthma. N Engl J Med.

[70] Castro M, Zangrilli J, Wechsler ME, Bateman ED, Brusselle GG (2015). Reslizumab for inadequately controlled asthma with elevated blood eosinophil counts: results from two multicentre, parallel, double-blind, radomised, placebo-controlled, phase 3 trials. Lancet Respi Med.

